# CUSHING'S SYNDROME CAUSED BY AN ADRENOCORTICAL CARCINOMA AFTER A BARIATRIC SURGERY: CASE REPORT

**DOI:** 10.1590/S0102-6720201500S100024

**Published:** 2015-12

**Authors:** Kátia Elisabete Pires SOUTO, Daniela Aline PEREIRA, Mauricio Jacques RAMOS, Alberto Salgueiro MOLINARI, Daniel de Carvalho DAMIN

**Affiliations:** 1 Endocrinology; 2 Digestive Surgery Services, Nossa Senhora da Conceição Hospital; ³Surgery Postgraduate Program, Hospital de Clinicas, Federal University of Rio Grande do Sul, Porto Alegre, RS, Brazil

## INTRODUCTION

Interest in the study of adrenocortical carcinoma has greatly increased in the last 12 years. In the last decade, more medical articles about this subject have been published than in the last 50 years^1^. Adrenal carcinoma is a rare and aggressive neoplasia and the affected patients have less than a 50% chance of survival in five years. In the metastatic disease, the survival rate is less than 15%^2^. It has an incidence of 0.7 to 0.2 per 1 million people each year with bimodal distribution and it is predominant in females^2^. The incidence is shown to be higher in children from the South of Brazil due to environmental and genetic factors^3^^,^ 4. It represents 3-6% of all carcinomas^3^. 

The purpose of this report is to relate a patient with morbid obesity that underwent bariatric surgery and after that developed the Cushing's syndrome caused by an adrenocortical carcinoma.

## CASE REPORT

A 54 years old patient was admitted to the endocrinology unit in February 2012. She had a 3-month history of acne, hirsutism, muscle weakness in her lower limbs, increasing levels of hypertension and diabetes mellitus imbalance. The physical exam revealed a rounded face, clavicular fatness, a fatty hump, BP= 200X100 mmHg, BMI=32.4 kg/m². She had a gastric bypass due to morbid obesity in 2009, with BMI=52,47 kg/m². Hormonal data were consistent with CS- ACTH independent (Table 1).

**TABLE 1:** - Laboratorial and weight evolution during the patient's follow-up

	**February 2012**	**May 2012**	**August 2012**	**March 2013**	**References**
ACTH	**<** 5.0	33.2	**<** 5.0	**<** 5.0	<46 pg/ml
DHEA-S	1410		248.6		19-205 u/dl
Androstenedione	>10	0.32	4.61	>10	0.40-3.0 ng/ml
17 -alpha-OH-progesterone	2.14		0.23	297ng/dl **	0.19 -1.0 ng/ml
SHBG	45.60	128.2	56.20		18-114 nmol/l
Free testosterone	6.78	0.05	0.48	23.75	0.19 - 2.06 ng/dl
Total testosterone	6.55		0.53		0.06-0.82 ng/ml
Urinary free cortisol	224.1	18.8	90.9		10-90 ug/24 h
LDDST* cortisol	31.87		9.75	15.23	
LH	<0.1		19.2		1.7 - 8.6mUl/ml

DHEA-S=plasma dehydroepiandrosterone sulfate; *LDDST=low dose dexamethasone suppression test; **change in the ng/dl method (VR: 59-344 ng/dl)

**FIGURE 1:**
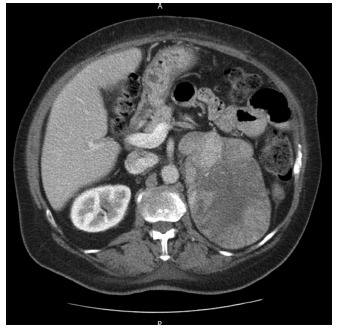
- Abdominal tomography showing adrenal tumor

Abdominal computed tomography showed an expanding lesion in the left adrenal area of 13.0X9.0 cm, with irregular contour lines and heterogeneous caption of contrast (Figure 1).

**FIGURE 2:**
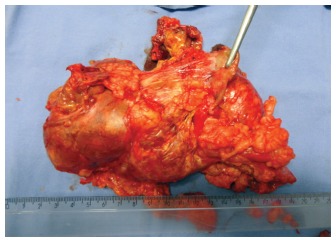
- Resected adrenal tumor

She was submitted to a left adrenalectomy and partial nephrectomy in April 2012. Her anatomopathological exam result was 630 g, size 21X10X 5.0 cm (Figure 2).

The pathologic findings were: 15 mitosis in 50 high power fields and the nuclear pleomorphisms was present; it showed the presence of confluent necrosis and capsular invasion; Ki-67 index was 20% positive according to the immunohistochemical criteria **;** TNM tumor stage was 3-0-0. 

**FIGURE 3:**
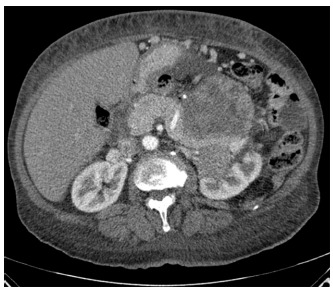
- Adrenal mass

There was regression of clinical and laboratorial alterations related to the Cushing's syndrome. She had a follow-up as an outpatient and used mitotane 2 g per day. After seven months, abdominal and thoracic tomography showed recurrence of the tumor and metastasis in the lungs (Figure 3).

As there was no satisfactory response from mitotane, cisplatin and etoposide, her clinical situation worsened and she died 13 months after having had an adrenalectomy. The patient's daughter has agreed in writing to allow the publication of this study.

## DISCUSSION

Brazil is the country with the second highest numbers of bariatric surgeries and it has the highest number of bariatric surgeons in the world^5^. Although Cushing syndrome is a rare cause of obesity, it is supposed that some of the patients that have had bariatric surgery have it. There are few studies about it in patients with morbid obesity^2^. Cushing is a disease difficult to diagnose and many times its diagnosis happens too late^6^. When hypercortisolism appears, it can be an ACTH - dependent Cushing's syndrome (70 to 80%) or ACTH - independent caused by adrenocortical tumors or hyperplasia adrenal^7^^,^ 8. It is more prevalent in high risk populations such as obese people with poorly controlled diabetes and its incidence ranges between 3.3 to 5.8% in published studies 8. Screening for Cushing before bariatric surgery has been suggested in order to avoid a procedure that could be lethal and irreversible as it has already been described^8^^,^ 9. There are reports about it over 9.33% in obese patients^10^. Approximately 60% of adrenal carcinomas are functioning and 70% are manifested by Cushing syndrome, associated or not to virilization. Virilization and DHEA-S high levels are more associated to malign tumors^11^. In those tumors, the symptoms associated to the glucocorticoids excess develop fast (from 3 to 6 months)^2^. Patients with higher recurrence risk present a high rate of malignity in the histology (Ki67 staining of >10% of tumor cells, >20 mitotic figures per 50 high power fields) no matter what the tumor size and the vascular and capsular invasion^12^. Ki67 is the most powerful detector of the located or advanced disease and it can guide the treatment^1^. Adrenal carcinomas grow very fast and very often they present metastasis in the lungs and liver^11^. This patient's adrenalectomy did not show evidence of metastasis. However, after seven months she had a recurrence of the disease and there was lung metastasis. This case highlights the fact that patients with morbid obesity should be investigated in regard to other endocrinal diseases. Future studies are needed to evaluate the true Cushing syndrome prevalence in this population^10^. It is believed that this is the first case reported in the literature that refers functioning adrenocortical carcinoma which was diagnosed after bariatric surgery. The authors emphasize the importance of having an endocrinologist in the surgical team, who also diagnoses and treats Cushing syndrome in morbidly obese patients.
